# Impact of post-transplant cyclophosphamide and anti-thymocyte globulin on immune reconstitution in MUD allo-HCT

**DOI:** 10.3389/fimmu.2026.1820251

**Published:** 2026-04-28

**Authors:** Giulia Furnari, Verena Wais, Anna Francesio, Katrin Strauss, Simona Piemontese, Jacqueline Schnell, Panagiota Gianni, Raffaella Greco, Frank Stegelmann, Annalisa Ruggeri, Andrea Assanelli, Donald Bunjes, Fabio Ciceri, Hartmut Döhner, Maria Teresa Lupo-Stanghellini, Elisa Sala

**Affiliations:** 1 Università Vita-Salute San Raffaele, Milan, Italy; 2 Hematology and Bone Marrow Transplantation Unit, San Raffaele Scientific Institute IRCCS, Milan, Italy; 3 Department of Internal Medicine III, University Hospital of Ulm, Ulm, Germany

**Keywords:** anti-thymocyte globulin, immune reconstitution, infections, matched unrelated donor, post-transplant cyclophosphamide, ATG, PTCY

## Abstract

Insufficient immune reconstitution (IR) is a major determinant of morbidity and mortality after allogeneic hematopoietic cell transplantation (allo-HCT). Strategies for graft-versus-host disease (GVHD)-prophylaxis, such as anti-thymocyte globulin (ATG) and post-transplant cyclophosphamide (PTCy), modulate immune recovery, but their effects on IR in matched unrelated donor (MUD) allo-HCT remain incompletely defined. In this retrospective bi-centric study, we analyzed patients with myeloid malignancies undergoing MUD allo-HCT who received ATG or PTCy per center policy. Longitudinal IR and clinical outcomes were assessed. IR was defined as sustained recovery of CD3+CD4+ cells >200/μl and CD19+ cells >50/μl. The impact of GVHD-prophylaxis (ATG vs PTCy) on IR dynamics was explored. A total of 252 patients were included. By day +365, 16.7% achieved IR, which was independently associated with superior OS (HR 0.39, 95% CI 0.17–0.90; p=0.026) and lower TRM (HR 0.08, 95% CI 0.01–0.63; p=0.017). In multivariable competing-risk analyses, younger donor age (sHR 0.97, 95% CI 0.94–1.00; p=0.037) and PTCy (sHR 0.49, 95% CI 0.27–0.84; p=0.01) were associated with higher probability of IR by month +18. The association between PTCy and IR was attenuated after adjusting for therapy-requiring acute or chronic GVHD, which independently delayed IR (HR 0.31, 95% CI 0.20–0.48; p<0.001). ATG and PTCy showed distinct IR trajectories: ATG associated with earlier NK expansion, PTCy led to enhanced adaptive T- and B-cell recovery from day +100. IR strongly predicted survival, independently of GVHD-prophylaxis. Prospective studies are warranted to better define determinants of IR after MUD allo-HCT in the PTCy era.

## Introduction

Allogeneic hematopoietic cell transplantation (allo-HCT) remains the only potentially curative strategy for many patients with high-risk myeloid malignancies. Despite major advances in donor selection, conditioning regimens, and supportive care, transplant-related morbidity and mortality continue to represent a significant clinical burden, accounting for at least 20% of overall mortality within 3 years after transplantation (1). Beyond graft-versus-host disease (GVHD), a delayed and dysfunctional immune reconstitution (IR) is a major determinant of infectious complications, non-relapse mortality (NRM), and long-term outcomes following allo-HCT (2–4).

The kinetics and quality of immune recovery after allo-HCT are shaped by multiple interacting factors, including recipient age, thymic function, conditioning intensity, donor matching, graft source, post-transplant immunosuppression and the occurrence of acute or chronic GVHD (5, 6). In particular, strategies for *in vivo* T-cell depletion used for GVHD-prophylaxis—such as anti-thymocyte globulin (ATG) or post-transplantation cyclophosphamide (PTCy)—play an important role in shaping IR.

While the dynamics and clinical correlates of immune recovery following matched unrelated donor (MUD) allo-HCT are not fully characterized, the risk factors influencing IR, particularly the role of immunosuppressive strategies for GVHD-prophylaxis, remain a topic of ongoing investigation.

In MUD allo-HCT, ATG and PTCy represent indeed two widely adopted approaches for *in vivo* modulation of donor T cells and both strategies are recommended as options in the setting of GVHD-prophylaxis by current international guidelines (7). Despite their shared clinical goal of preventing GVHD, ATG and PTCy exert their immunomodulatory effects through distinct biological mechanisms and at different time points after allo-HCT, which could have potential implications for the dynamics of IR. ATG induces broad lymphocyte depletion through antibody-mediated mechanisms (8), whereas PTCy preferentially targets proliferating T cells early after graft infusion, with relative preservation of non-proliferating and regulatory immune subsets (9). Understanding how these approaches influence the timing and dynamics of IR, as well as their association with infectious complications, is essential for optimizing post-transplant outcomes.

Here we report a retrospective bi-center study analyzing adult patients undergoing first allo-HCT from 10/10 MUD for myeloid malignancies and describing the incidence of IR and related infectious complication during the post-transplantation follow-up. Our aim was to identify potential risk factors for delayed immune recovery, with particular emphasis on the impact of different GVHD-prophylaxis strategies (ATG vs PTCy).

## Materials and methods

### Data collection and eligibility criteria

This retrospective bi-center observational study included adult patients with myeloid malignancies undergoing first allo-HCT from a 10/10 MUD between 2019 and 2023 at two European transplant centers: San Raffaele Hospital (Milan, Italy) and Ulm University Hospital (Ulm, Germany). The study protocol was approved by the local ethics committees. The study was conducted in accordance with the Declaration of Helsinki and Good Clinical Practice guidelines. All patients provided informed consent for pseudo-anonymous data collection within non-interventional institutional registries and analysis in scientific context. Inclusion criteria were (1) confirmed diagnosis of myeloid malignancy with an indication for allo-HCT, including acute myeloid leukemia (AML), myelodysplastic syndrome (MDS), myeloproliferative neoplasm (MPN) and myelodysplastic/myeloproliferative neoplasms (MDS/MPN), as previously defined (10); (2) adult (age ≥ 18 years); (3) first allo-HCT; (4) receiving transplantation from a 10/10 MUD; (5) *in-vivo* T cell depletion with ATG (selected GVHD-prophylaxis at the Ulm center per institutional policy) or PTCy (selected GVHD-prophylaxis at the Milan center per institutional policy). All patients underwent a pretransplant assessment of organ function. Comorbidity scores as the European Society for Blood and Marrow Transplantation (EBMT) risk score were calculated as described previously (11).

### Conditioning regimens, GVHD-prophylaxis and treatment, and other definitions

Transplant conditioning intensity was defined according to the Transplant Conditioning Intensity (TCI) score (12). GVHD-prophylaxis strategy was determined by institutional practice.

ATG (Grafalon^®^, Neovii, Switzerland) was given at a total dose of 30 or 60 mg/kg, depending on the risk profile of the underlying disease and on the intensity of conditioning regimen. ATG doses were fractionated between days - 4 to -2 before allo-HCT and was given in combination with a calcineurin inhibitors (CNI) from day -3 and mycophenolate mofetil (MMF) from day 0 to day +65.

PTCy was predominantly administered at 50 mg/kg/day on days +3 and +4. Patients considered high risk according to the CARE-BMT risk score (13) received as a reduced regimen a total dose of 80 mg/kg. PTCy was combined with sirolimus from day +5 and with MMF from day +5 to day +28. In both centers, tapering of immunosuppressive therapy generally commenced between day +60 and +90 in patients without evidence of GVHD. The kinetics of drug discontinuation were strictly risk-adapted, aiming for earlier cessation in patients with high-risk disease to favor graft-versus-leukemia effects, while ensuring clinical stability. In case of GvHD onset, both acute and chronic, first-line treatment consisted of systemic corticosteroids (prednisone or methylprednisolone at 12 mg/kg/day in acute GvHD, prednisone 0.5–1 mg/kg/day in chronic GvHD) at both institutions according to EBMT recommendation (7). For steroid-refractory cases, ruxolitinib represented the preferred second-line strategy for both acute and chronic GVHD, based on the results of the phase III REACH2 and REACH3 trials (14, 15) and EBMT recommendations (7). In patients with contraindications to ruxolitinib (e.g., severe cytopenias) or refractory disease, alternative approaches were utilized based on clinical judgment, including extracorporeal photopheresis (ECP), TNF-alpha inhibitors, or ROCK2 inhibitors (16).

Considering the anti-infectious prophylaxis, all patients received trimethoprim-sulfamethoxazole for Pneumocystis jiroveci prophylaxis, acyclovir for herpesvirus prophylaxis, and letermovir for cytomegalovirus (CMV) prophylaxis in seropositive patients. Azoles were administered as antifungal prophylaxis. Patients were screened weekly at least for the first six months after allo-HCT for CMV and Epstein-Barr virus (EBV) by polymerase chain reaction (PCR) on blood samples.

Recipient/donor CMV serostatus was classified into high-risk (recipient [R]+/donor [D]+ or R+/D–), intermediate-risk (R–/D+), and low-risk (R–/D–) categories (17, 18).

Overall survival (OS) was defined as the number of days from transplant to death. Transplant-related mortality (TRM) was defined as death resulting from complications directly attributable to the hematopoietic cell transplantation (HCT) process, including but not limited to infections, graft-versus-host disease (GVHD), organ toxicity, and other transplant-related factors, rather than from relapse of the underlying malignancy.

### Monitoring of immune reconstitution

Flow cytometric immunophenotyping for monitoring of IR was performed from patient’s peripheral blood samples on day (d)+30, d+100, d+180 and d+365 post-transplant, and afterwards every 3 months until IR. For T cells, CD3+ and subsets CD3+CD4+ and CD3+CD8+ were analyzed. NK cells were defined as CD3-, CD56+ and CD16+, and for B cells as CD19 +. IR was defined as the detection in two consecutive measurements of CD3+CD4+ T- cells > 200 cells/μl and CD19+-B-cells > 50 cells/μl, as previously described (19, 20).

### Statistical analysis

Categorical variables were summarized using frequencies and percentages, and continuous variables using medians with interquartile ranges. Percentages are calculated excluding missing values.

IR dynamics and immune-correlated clinical events were primarily analyzed descriptively over time. Time-to-event outcomes, such as IR and TRM, were estimated using the cumulative incidence, accounting for competing risks where appropriate. Death without the event of interest was treated as a competing event when applicable.

Overall survival (OS) was estimated using the Kaplan–Meier method (21). Extended Cox models (22) were used to assess the impact of IR on overall survival. For exploratory analyses of factors associated with delayed IR, multivariable Fine–Gray sub-distribution hazard models were constructed to evaluate predictors of IR within 18 months post-transplant, considering death before IR as a competing event. Covariates included: EBMT risk score, conditioning intensity (TCI), donor age, CMV serostatus matching between donor and recipient, and GVHD-prophylaxis platform (ATG versus PTCy). In complementary analyses, clinically relevant GVHD (defined as acute GVHD of grade ≥ II according to MAGIC (23) or chronic moderate/severe GVHD according to NIH (24)) was modelled as a time-dependent covariate in cause-specific Cox regression to assess its association with time to IR.

All statistical analyses were conducted using R statistical software (version 4.5.1, R Foundation for Statistical Computing, Vienna, Austria).

## Results

### Patient and transplant characteristics

A total of 252 patients met the inclusion criteria and were included in the analysis.

The study population comprised both male (59.5%) and female (40.5%) patients, with a median age at allo-HCT of 61 years (IQR 54–66). Underlying diagnoses comprised AML (156 patients, 61.9%), MDS (44 patients, 17.5%), MPN (34 patients, 13.5%), and MPN/MDS (18 patients, 7.1%).

Disease status at transplantation included mostly complete remission (213 patients, 84.5%). Median donor age was 30 years (IQR 25–37). High-risk CMV serostatus was observed in the majority of patients (n=144, 57.8%). Considering the intensity of conditioning, most of the patients received a regimen falling into the intermediate category according to the TCI score (n= 175, 69.4%). The median infused CD34^+^ cell dose was 7.0 × 10^6^/kg (IQR 5.5–7.87). An ATG-based GVHD-prophylaxis was used in 70% of the cases (n=177 patients), while 75 patients (30%) received PTCy. Patient and transplant characteristics are reported in **Table 1**. The cumulative incidence of acute GvHD grade II-IV at day +100 after allo-HCT was 52.2% (95% CI 46%–58.%), 66.7% (95% CI, 59.7%-73.6%) in the ATG+CNI subpopulation and 17.8% (95% CI, 8.9%-26.6%) in the PTCy+sirolimus patients. The cumulative incidence of moderate to severe chronic GvHD at 1 year after allo-HCT was 20% (95% CI 15%-25%), with respectively 20% (95% CI 14%-25.9%) in the ATG+CNI patients and 20% (95% CI 10.5%-29.4%) in the PTCy+ sirolimus ones.

**Table 1:** Patient and transplant characteristics.

Variables	Total (n=252)	ATG (n=177)	PTCy (n=75)
Patient characteristics			
Gender, n (%)			
Male	150 (59.5%)	106 (60%)	44 (59%)
Female	102 (40.5%)	71 (40%)	31 (41%)
Age at transplant, median (IQR)	61 (54 – 66)	61 (55 – 66)	60 (51 - 66)
Disease, n (%)			
AML	156 (61.9%)	106 (60%)	50 (67%)
MDS	44 (17.5%)	31 (18%)	13 (17%)
MPN	34 (13.5%)	25 (14%)	9 (12%)
MDS/MPN	18 (7.1%)	15 (8.5%)	3 (4%)
EBMT risk score			
Low (0-2), n (%)	20 (8.1%)	15 (8.7%)	5 (6.8%)
Intermediate (3-4), n (%)	139 (56.5%)	83 (48%)	56 (76.7%)
High (5-7), n (%)	87 (35.4%)	75 (43.4%)	12 (16.4%)
Missing	6	4	2
Disease status at HSCT, n (%)			
CR	213 (84.5%)	155 (88%)	58 (77%)
Not in CR	39 (15.5%)	22 (12%)	17 (23%)
Donor characteristics			
Donor age, median (IQR)	30 (25 – 37)	31 (25 – 37)	28 (24 - 37)
Sex D/R, n (%)			
Female/Male	28 (11.1%)	20 (11%)	8 (11%)
Others	224 (88.9%)	157 (89%)	67 (89%)
CMV serostatus (R/D), n (%)			
High risk (+/+,+/-)	144 (57.8%)	79 (44.6%)	65 (90.3%)
Intermediate risk (-/+)	26 (10.4%)	25 (14.1%)	1 (1.4%)
Low risk (-/-)	79 (31.7%)	73 (41.2%)	6 (8.3%)
Missing	3	0	3
Transplant characteristics			
Transplant conditioning intensity median (range)	3.5 (1.5-5)	3.5 (1.5 - 5)	3.5 (1.5 – 5.5)
TCI low, n (%)	12 (4.8%)	1 (0.6%)	11 (15%)
TCI intermediate, n (%)	175 (69.4%)	131 (74%)	44 (59%)
TCI high, n (%)	65 (25.8%)	45 (25%)	20 (27%)
CD34+ cells x10^6/kg, median (IQR)	7 (5.5 – 7.87)	6.91 (5.44 - 8.57)	6.48 (5.63 – 7.02)
GvHD Prophylaxis, n (%)			
CNI + ATG	177 (70.2%)	177 (100%)	0 (0%)
mTOR + PTCy	75 (29.8%)	0 (0%)	75 (100%)

IQR, interquartile range; AML, acute myeloid leukemia; MDS, myelodysplastic neoplasm; MPN, myeloproliferative neoplasm; MDS/MPN, myelodysplastic/myeloproliferative neoplasms; CR, complete remission; CMV, cytomegalovirus; TCI, transplant conditioning intensity; GVHD, graft-versus-host disease; ATG, anti-thymocyte globulin; CNI, calcineurin inhibitor; MMF, mycophenolate mofetil; PTCy, post-transplant cyclophosphamide.

### Cumulative incidence of immune reconstitution and impact on outcomes

By day 180 after allo-HCT, the cumulative incidence of IR was 3.6% (95% CI, 1.6–6.1). By day 365, it increased to 16.7% (95% CI, 12.1–21.6), and reached 25.4% (95% CI, 20.1–31.2) by 18 months. The median time to IR was not reached within the first year of follow-up.

**Figure 1:**
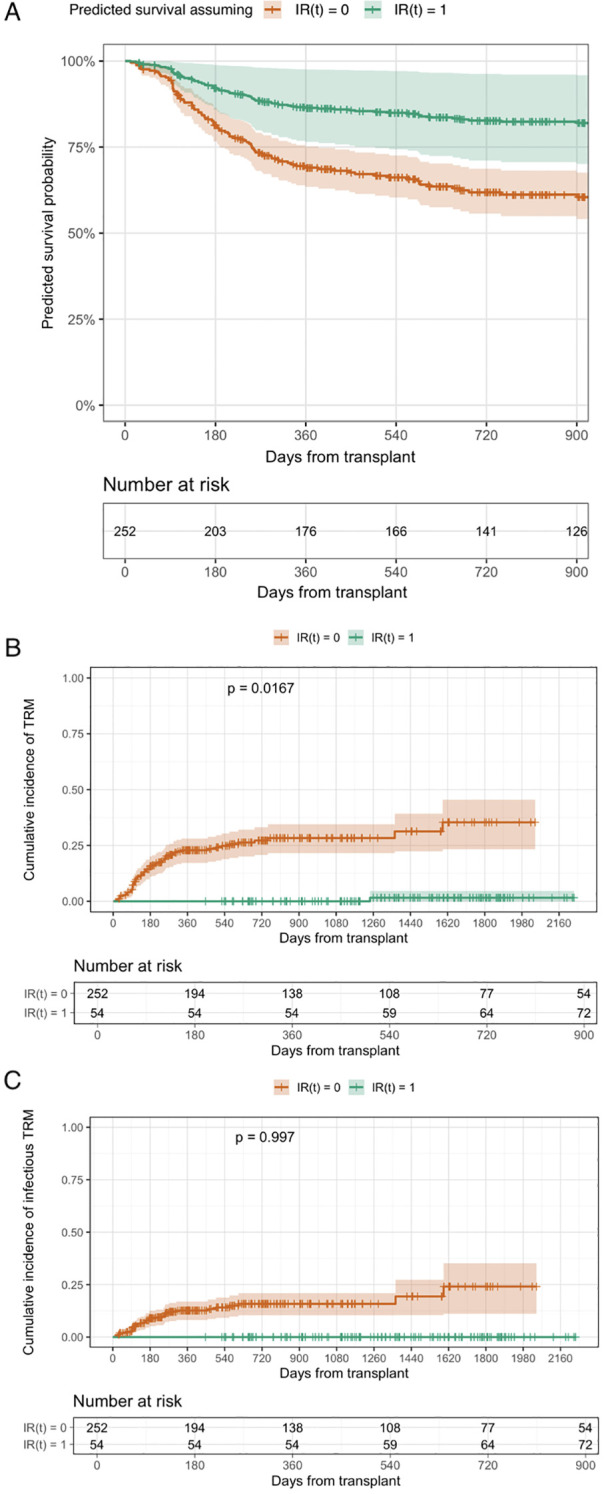
**(A–C)** Time-dependent effect of immune reconstitution on OS, TRM and infectious TRM. Predicted survival probabilities derived from a Cox proportional hazards model including immune reconstitution as a time-dependent covariate. Curves represent model-based survival estimates assuming immune reconstitution status IR(t)=1 versus IR(t)=0 at a given time point. These curves do not represent Kaplan–Meier estimates or observed patient groups.

The 2y OS of the entire cohort was 67.0% (95% CI 61.3–73.1%). When modeled as a time-dependent covariate, IR acquisition was associated with improved OS (hazard ratio [HR] 0.39, 95% CI 0.17–0.90; p = 0.026) (**Figures 1a, b**). At 2 years after allo-HCT, the cumulative incidence of transplant-related mortality was 23.7% (95% CI, 18.3–29.2). Similarly, IR acquisition was associated with a lower hazard of TRM (HR 0.08, 95% CI 0.01–0.63; p = 0.017). No infectious deaths occurred after the achievement of IR (**Figure 1c**).

### Determinants of immune reconstitution

In multivariable competing-risk analysis, GVHD-prophylaxis platform was independently associated with IR. Compared with PTCy-based prophylaxis, ATG was associated with a significantly lower cumulative incidence of IR (sHR 0.48, 95% CI 0.27–0.84; p=0.010). Increasing donor age (analyzed as a continuous variable given the narrow IQR: 25–37) was likewise associated with reduced probability of IR (sHR 0.97 per year, 95% CI 0.94–0.998; p=0.037), whereas recipient CMV seropositivity was associated with a markedly higher cumulative incidence of IR (sHR 3.50, 95% CI 1.53–7.97; p=0.003). The EBMT-risk score and TCI did not show a significant impact on IR.

Therapy-requiring acute (grade II-IV) and chronic (moderate to severe) GVHD was accounted for as a time-dependent variable, its occurrence was strongly associated with delayed IR (HR 0.31, 95% CI 0.20–0.48; p<0.001). In this model, the association between prophylaxis platform and IR was attenuated and no longer statistically significant (HR 0.80; p=0.45).

### Immune reconstitution dynamics

To explore the biological dynamics underlying the association between GVHD-prophylaxis platform (ATG and PTCy) and IR, we examined the longitudinal recovery of lymphocyte subsets during the first 360 days after transplantation. Overall, all major lymphocyte populations exhibited progressive recovery over time.

Focusing on the group of patients who received ATG, we observed an early expansion of the NK-cell compartment, with a median absolute count of 126 cells/µl (range 63–268) at day +30 and 235 cells/µl (range 138–359) at day +100 after allo-HCT. In contrast, patients receiving PTCy showed markedly lower NK-cell counts at day +30, with a median of 3 cells/µl (range 1–46), increasing to 98 cells/µl (range 70–150) at day +100, indicating a slower early recovery kinetics.

**Figure 2:**
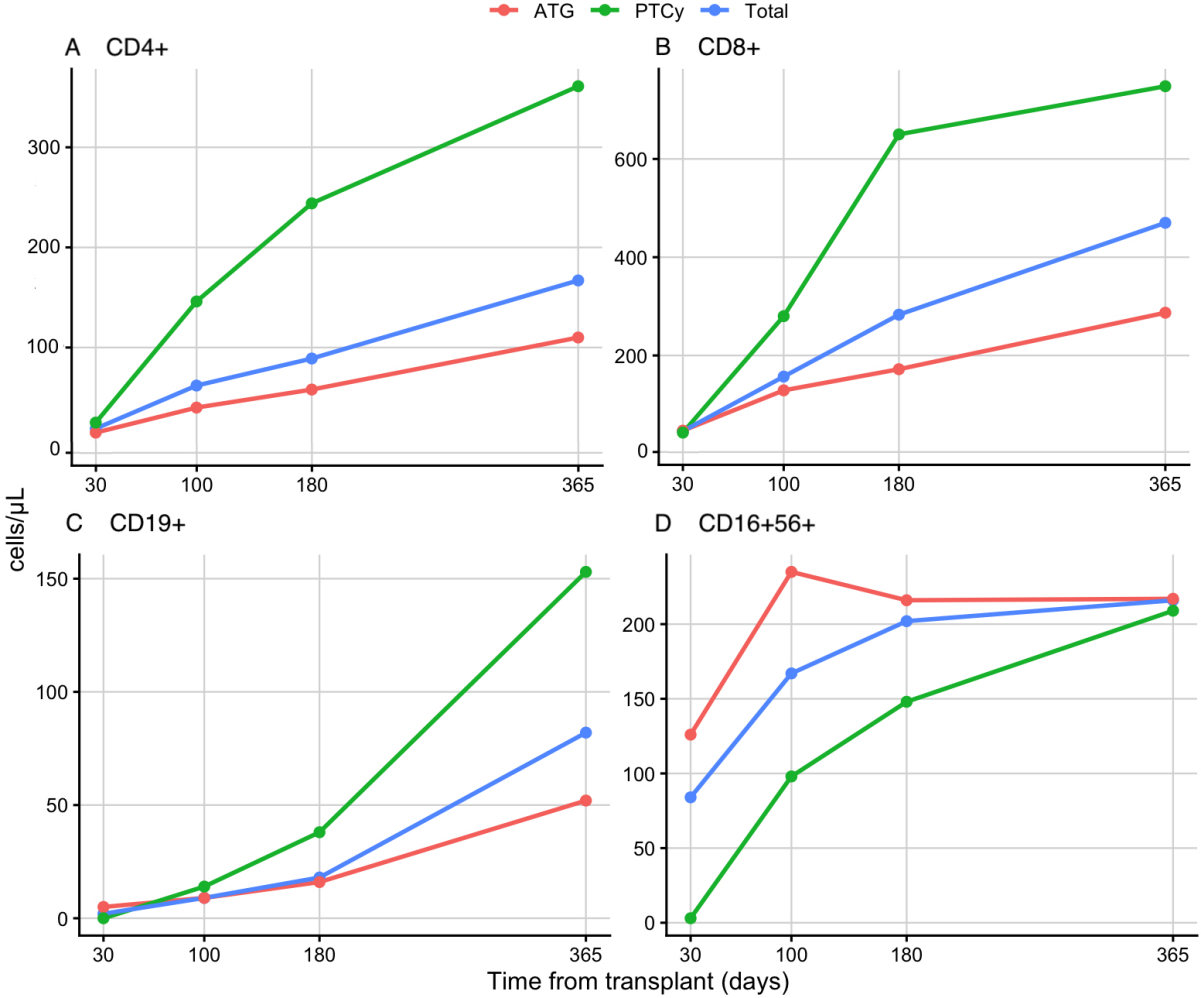
Immune reconstitution kinetics after allo-HCT. Platform-specific trajectories are displayed to provide clinical context and illustrate heterogeneity in immune reconstitution, without formal comparative testing. CD4+ **(A)** and CD8+ **(B)** T-cell reconstitution showed a progressive and sustained increase over time in patients receiving PTCy. B-cell recovery [CD19+, **(C)**] was delayed in both cohorts early after transplant, and accelerated after PTCy from day +180 onward. NK-cell counts [CD16 + 56+, **(D)**] were higher in the ATG group during the early phase and became equivalent between groups at one year.

Conversely, the CD3+ T-cell compartment (both CD3+CD4+ and CD3+CD8+ subsets) demonstrated a faster and more pronounced increase over time in patients receiving PTCy compared to those having ATG. Although early post-transplant (day +30) CD3+CD4+ and CD3+CD8+ T-cell counts were low in the PTCy group (median 25 cells/µl [range 9–87] and 43 cells/µl [range 5–174], respectively), a robust and sustained reconstitution became evident from day +100 through day +365, with higher absolute counts than those observed in the ATG group (**Figure 2**). A similar pattern was observed for CD19+ B-cell reconstitution (**Figure 2**).

### Infectious events

We then focused on the incidence of severe infections, especially during the early follow-up after allo-HCT. Infectious complications requiring hospitalization within the first 6 months after transplantation were observed in 77 out of 252 patients (30.6%). All these events occurred before documented IR.

Bacterial (36.4%) and viral infections (33%) accounted for the majority of events, while fungal complications had a lower incidence (11.4%). In 19.3% of cases the infectious etiology could not be clearly identified.

Within patients receiving ATG, 64 out of 177 patients (36.2%) experienced at least one severe infection requiring hospitalization by day +180, most frequently of bacterial origin (36.6%), followed by viral (29.6%) and fungal one (12.7%). Thirteen out of 75 patients receiving PTCy (17.3%) experienced severe infections requiring hospitalization within the first 6 months after allo-HCT. Viral infections accounted for the largest proportion of events (47.1%), followed by bacterial infections (35.3%), whereas fungal complications were less frequent (5.9%).

Focusing of viral infections, we further examined the occurrence and clinical spectrum of CMV reactivations. Overall, within the first post-transplant year CMV reactivation of any grade was observed in 62 of 252 patients (24.6%). Of these, transient viremia occurred in 9 patients (3.6%), reactivation requiring pre-emptive therapy in 46 (18.2%), and CMV disease in 7 patients (2.8%). CMV reactivations were observed both in patients receiving ATG (51 patients) and PTCy (11 patients). Regarding EBV, reactivations requiring treatment (Rituximab) occurred in 36 out of 252 patients (14.3%) over the entire follow-up, notably all receiving ATG as GVHD-prophylaxis.

## Discussion

In this retrospective and observational bi-center study of a large (n=252) cohort of consecutive adult patients undergoing allo-HCT from 10/10 MUD for myeloid malignancies between 2019 and 2023, we provide a comprehensive description of IR dynamics over the first post-transplant year and explore their clinical relevance with respect to infectious complications and survival.

As surrogate marker for achieved immune competence we used the detection in two consecutive measurements of a CD3+CD4+ T- cells absolute count > 200/μl and CD19+-B-cells > 50/μl, based also on previous reports (25–27).

Our study demonstrates a strong association between the onset of IR and improved survival post-allo-HCT. IR was associated with improved OS (HR = 0.39, 95% CI 0.17–0.90, p = 0.026), corresponding to a 60% reduction in the hazard of death. Notably, achievement of IR was associated with a 92% lower risk of TRM (HR = 0.08, 95% CI 0.01–0.63, p = 0.017). Strikingly, no infectious transplant-related deaths occurred after IR had been achieved in our cohort.

Considering that the median time to IR was not reached within the first year of follow-up, we aimed to identify clinical factors associated with delayed immune recovery. In multivariable competing risks analysis, one of the most relevant findings was that PTCy-based GVHD-prophylaxis appeared to be associated with a higher CI of IR at 18 months after allo-HCT in the setting of matched unrelated donor. These findings are in line with previous studies evaluating IR in the setting of haploidentical allo-HCT and comparing ATG versus PTCy platforms (28, 29). In a recent published meta-analysis on IR after haploidentical allo-HCT (30), Jin et al. provide literature-based evidence that PTCy accelerates lymphocyte reconstitution, significantly reducing the risk of fungal infection, and improving the OS, LFS and GRFS as compared with ATG. In the setting of matched unrelated donor allo-HCT only few studies comparing the two platforms are available to our knowledge (31, 32). Kerbauy and colleagues described a later B-, T- and NK-cell reconstitution in patients receiving PTCy compared to ATG in a prospective cohort study analyzing overall 36 patients (n=20 receiving ATG, n=16 receiving PTCy) in patients with different types of hematological malignancies and receiving in 52.7% of the cases (n=19) bone marrow as allo-HCT source (33).

However, in our study, the association between GVHD-prophylaxis and IR was attenuated after accounting for the occurrence of therapy-requiring acute or chronic GVHD, which was strongly associated with delayed immune recovery (HR 0.31, 95% CI 0.20–0.48, p<0.001). This attenuation likely reflects the close relationship between GVHD and the subsequent need for systemic immunosuppression, both of which substantially affect the kinetics of immune reconstitution. In our model, clinically significant GVHD may therefore capture the cumulative impact of post-transplant inflammatory events and the intensification of immunosuppressive therapy, two key determinants of immune recovery. Additionally, variability in outcomes may also reflect differences in GVHD incidence and in responsiveness to systemic immunosuppressive treatment, as suggested by recent studies (34, 35). We also observed an association between donor age and delayed IR, consistent with prior evidence suggesting that graft-intrinsic characteristics (5, 6) may influence immune recovery dynamics. Particularly, emerging evidences are suggesting how donor age deeply impacts allo-HCT outcomes and is associated with delayed IR. Furthermore, recipient CMV seropositivity was associated with a higher cumulative incidence of IR, in line with the idea that subclinical antigenic stimulation in seropositive recipients may enhance T-cell expansion and functional immune recovery (36).

In exploring the immune recovery patterns and dynamics, ATG was associated with early NK cell expansion (32, 37) and a more prolonged delay in T- and B-cell recovery, reflecting its broad and persistent lymphodepleting effects (38, 39). While the earlier NK-cell recovery in the ATG group provides a biological basis for early immunosurveillance and potential protection from early viral infections, its clinical impact must be interpreted cautiously, as it did not preclude specific viral complications like EBV reactivation in our cohort. On the other side, PTCy exhibited a more gradual but sustained reconstitution of adaptive immune subsets (both CD3+CD4+/CD3+CD8+-T-cells and CD19+-B-cells), consistent with its immunomodulatory effects (40, 41). This finding adds to the growing body of literature that compares IR dynamics in different GVHD-prophylaxis strategies, especially ATG versus PTCy, in haploidentical setting, as well as in mismatched and matched unrelated donor allo-HCT context (31, 33, 42, 43).

Finally, we explored the relationship between IR and infectious complications. Severe infections requiring hospitalization within the first 180 days after allo-HCT occurred in nearly one-third of patients, with no such events in those who achieved early IR. This finding should be interpreted cautiously given the descriptive nature of this sub-analysis but is consistent with the knowledge that even partial restoration of adaptive immunity may confer clinically relevant protection against severe infections (44, 45). Analysis of infectious etiologies revealed that bacterial and viral infections accounted for the majority of events (respectively, 36.4% and 33%), whereas invasive fungal infections were less frequent (11.4%). Etiologic patterns were reported descriptively by GVHD-prophylaxis platform to provide clinical context; no formal comparisons were performed, and no causal inferences are intended. Of note, EBV reactivation requiring Rituximab treatment occurred exclusively in patients receiving ATG-based GVHD-prophylaxis, a well-known effect (32) that biologically aligns with the prolonged depletion of EBV-specific memory CD8+ cytotoxic T-cell pools by ATG, which allows expansion of latently infected B cells (46).

Our study with its retrospective, real-life nature, has important limitations. Most notably, GVHD prophylaxis was allocated according to center practice, with complete overlap between treatment platform and transplant center (ATG used in one center and PTCy in another). As a result, treatment platform and center effects are fully confounded and cannot be disentangled, precluding adjustment for center effects. Consequently, the ATG and PTCy cohorts cannot be statistically compared, as any observed differences may reflect center-specific practices rather than the effect of the GVHD prophylaxis strategy itself. Accordingly, these findings should be interpreted cautiously, as potential center-specific biases including differences in supportive care cannot be entirely ruled out. In addition, factors such as center-specific baseline immunosuppressive therapy (CNI in association with ATG and sirolimus in association with PTCy), GVHD incidence, and subsequent intensification of immunosuppression, may have influenced both immune recovery and clinical outcomes. In addition, immune phenotyping was restricted to quantitative lymphocyte subsets and did not capture functional or antigen-specific immunity. Finally, we did not assess thymic output markers (e.g., TRECs), which could further characterize the quality of T-cell recovery at later time points.

Despite these limitations, our study provides clinically relevant insights on the IR and its dynamics based on a large population of patients with myeloid malignancies receiving allo-HCT from matched unrelated donor. Our data highlight that delayed combined T- and B-cell recovery is common after MUD allo-HCT and identify IR as a strong, independent prognostic marker of survival. Even in the setting of modern antiviral prophylaxis strategies, coordinated adaptive immune recovery remains essential for durable host protection and optimal post-transplant outcomes. These findings support systematic longitudinal immune monitoring as part of post-transplant care and suggest that strategies aimed at promoting timely immune recovery—while maintaining effective GVHD control—may translate into improved outcomes.

Prospective, multicenter studies specifically designed to compare GVHD-prophylaxis strategies within the same institutional framework and incorporating comprehensive immune profiling will be necessary to clarify the causal relationships between prophylactic platform, GVHD, immune reconstitution, and long-term survival. In addition, evolving strategies for GVHD management - including novel immunomodulatory approaches such as adoptive immunotherapy with regulatory T cells - may further influence immune recovery after allo-HCT and should be more granularly evaluated in future investigations. Until such data are available, our results should be considered hypothesis-generating, while underscoring the central role of coordinated adaptive immune recovery in determining post-transplant success.

## Data Availability

The raw data supporting the conclusions of this article will be made available by the authors, without undue reservation.
